# Corrigendum to “The Effect of Art Therapy and Music Therapy on Breast Cancer Patients: What We Know and What We Need to Find Out—A Systematic Review”

**DOI:** 10.1155/2021/9870102

**Published:** 2021-05-12

**Authors:** Justina Kievisiene, Rasa Jautakyte, Alona Rauckiene-Michaelsson, Natalja Fatkulina, Cesar Agostinis-Sobrinho

**Affiliations:** ^1^Faculty of Health Sciences, Klaipeda University, Herkaus Manto g. 84, Klaipeda 92294, Lithuania; ^2^Institute of Health Sciences, Faculty of Medicine, Vilnius University, M. K. Čiurlionio g. 21/27, Vilnius 03101, Lithuania

In the article titled “The Effect of Art Therapy and Music Therapy on Breast Cancer Patients: What We Know and What We Need to Find Out—A Systematic Review” [1], there was an error in Figure 1. The authors apologize for this error and confirm that it does not affect the results and the conclusions of the article. The corrected figure, as approved by the editorial board, is as follows.

## Figures and Tables

**Figure 1:**
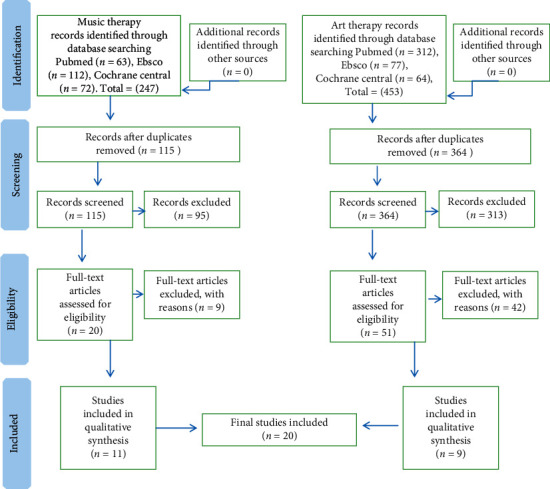
Flow of citations and articles through the phases of screening and eligibility evaluation.
